# Genopyc: a Python library for investigating the functional effects of genomic variants associated to complex diseases

**DOI:** 10.1093/bioinformatics/btae379

**Published:** 2024-06-18

**Authors:** Francesco Gualdi, Baldomero Oliva, Janet Piñero

**Affiliations:** Integrative Biomedical Informatics, Research Program on Biomedical Informatics (IBI-GRIB), Hospital Del Mar Medical Research Institute (IMIM), Department of Experimental and Health Sciences, Universitat Pompeu Fabra (UPF), C/ del Dr. Aiguader 88, Barcelona 08003, Spain; Structural Bioinformatics Lab, Research Program on Biomedical Informatics (SBI-GRIB), Hospital Del Mar Medical Research Institute (IMIM), Department of Experimental and Health Sciences, Universitat Pompeu Fabra (UPF), C/ del Dr. Aiguader 88, Barcelona 08003, Spain; Structural Bioinformatics Lab, Research Program on Biomedical Informatics (SBI-GRIB), Hospital Del Mar Medical Research Institute (IMIM), Department of Experimental and Health Sciences, Universitat Pompeu Fabra (UPF), C/ del Dr. Aiguader 88, Barcelona 08003, Spain; Integrative Biomedical Informatics, Research Program on Biomedical Informatics (IBI-GRIB), Hospital Del Mar Medical Research Institute (IMIM), Department of Experimental and Health Sciences, Universitat Pompeu Fabra (UPF), C/ del Dr. Aiguader 88, Barcelona 08003, Spain; Medbioinformatics Solutions SL, Barcelona, C/ rambla Cataluña 14, Barcelona 08007, Spain

## Abstract

**Motivation:**

Understanding the genetic basis of complex diseases is one of the main challenges in modern genomics. However, current tools often lack the versatility to efficiently analyze the intricate relationships between genetic variations and disease outcomes. To address this, we introduce Genopyc, a novel Python library designed for comprehensive investigation of how the variants associated to complex diseases affects downstream pathways. Genopyc offers an extensive suite of functions for heterogeneous data mining and visualization, enabling researchers to delve into and integrate biological information from large-scale genomic datasets.

**Results:**

In this work, we present the Genopyc library through application to real-world genome wide association studies variants. Using Genopyc to investigate the functional consequences of variants associated to intervertebral disc degeneration enabled a deeper understanding of the potential dysregulated pathways involved in the disease, which can be explored and visualized by exploiting the functionalities featured in the package. Genopyc emerges as a powerful asset for researchers, facilitating the investigation of complex diseases paving the way for more targeted therapeutic interventions.

**Availability and implementation:**

Genopyc is available on pip https://pypi.org/project/genopyc/.The source code of Genopyc is available at https://github.com/freh-g/genopyc. A tutorial notebook is available at https://github.com/freh-g/genopyc/blob/main/tutorials/Genopyc_tutorial_notebook.ipynb. Finally, a detailed documentation is available at: https://genopyc.readthedocs.io/en/latest/.

## 1 Introduction

The onset of complex disorders is influenced by a multitude of components that include lifestyle, diet, environmental and genetic factors. In the last decades genome wide association studies (GWAS) have emerged as a powerful tool to investigate the genetic architecture underlying complex diseases (Bush and Moore 2012). However, now that thousands of genetic loci have been successfully associated to numerous phenotypes, we are facing another challenge: the interpretation of these associations in the biological context. We are thus entering in the so called post-GWAS Era (Gallagher and Chen-Plotkin 2018). Understanding how genetic variants are translated into biological pathways remains a complex task (Edwards *et al.* 2013) that brought to the development of numerous approaches to interpret GWAS results [see (Uffelmann *et al.* 2021) for a comprehensive review of the type of analysis and tools].

One of the main pitfalls consists in handling and interpret the extensive amount of data required to perform these studies (Edwards *et al.* 2013). In response, a plethora of novel methodological approaches has emerged to address this knowledge gap (Mulder and Opap 2017). These techniques rely on the large-scale omics datasets and repositories available to researchers such as Gene Expression Omnibus (Edgar *et al.* 2002), the genotype—tissue expression project (GTEx) (Lonsdale 2013) and the Encode project (de Souza 2012). The enormous amount of data regarding genes and variants associated to diseases is collected in knowledge bases such as the GWAS Catalog (Sollis *et al.* 2023), and DisGeNET (Piñero *et al.* 2019) that offers a standardized integration from different sources. However, 90% of the genetic variation associated to complex diseases are noncoding type and a benchmark of method to interpret how they alter genes, perturb biological pathways and ultimately lead to disease is still missing (Li and Ritchie 2021). Moreover, the application and integration of different tools to analyze GWAS data lead to discordant results, thus an unbiased assessment of the methods available is still required (P**é**rez-Granado *et al.*  2022). An advancement in associating genes to noncoding variants has been made by the Open Target Genetics platform, which implemented a pipeline consisting of a machine learning model that uses heterogeneous features such as distance from variant to the gene, expression quantitative trait loci, chromatin conformation and variant effect predictor. This method outperformed the na_**ï**_ve distance-based methods in the prioritization of causal genes related to complex diseases loci (Mountjoy *et al.* 2021).

**Figure 1.**
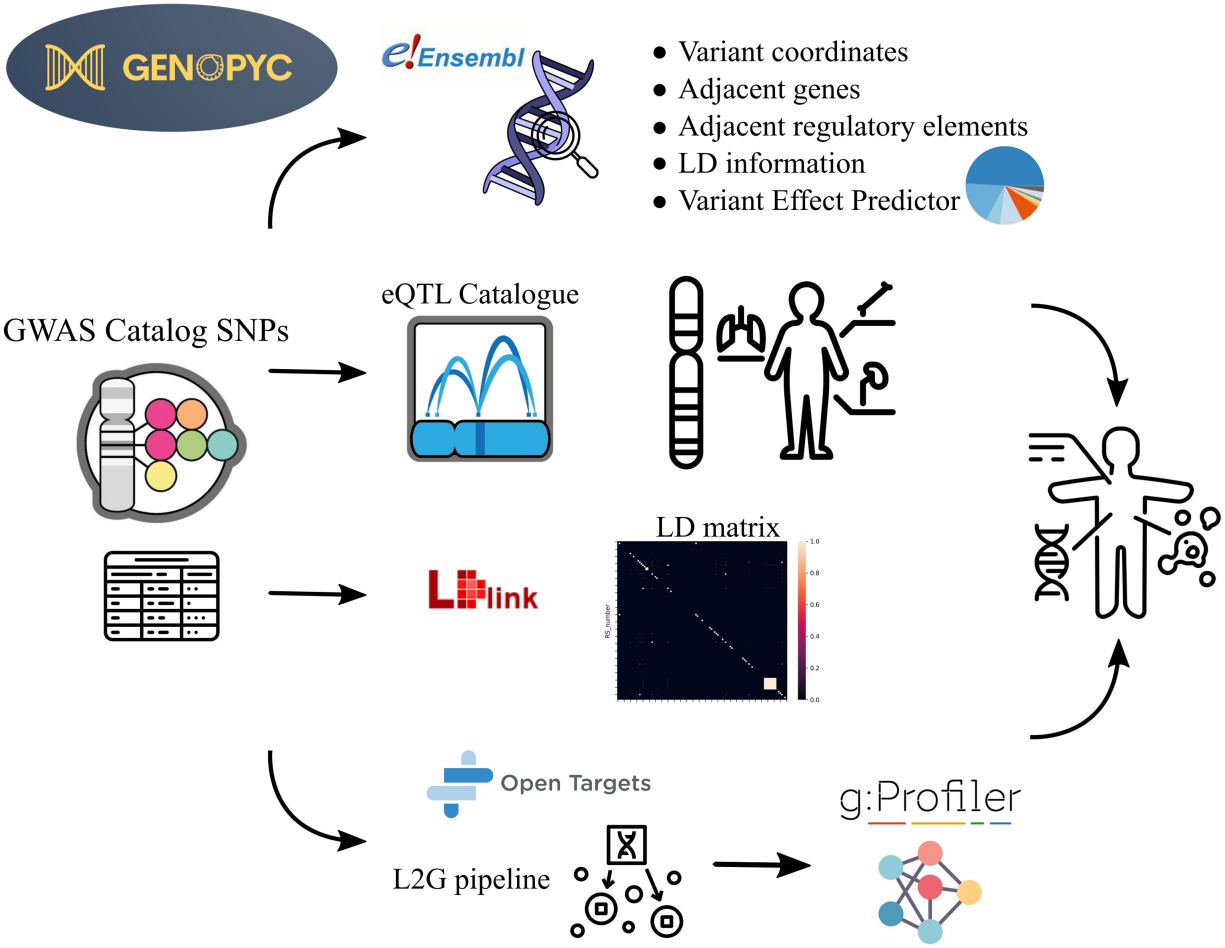
The main Genopyc features and knowledge bases accessed schematically represented. Variants associated with a specific trait are initially obtained from GWAS catalog and then subjected to various analyses, including examination of genomic context, LD features, eQTLs, VEP, and Locus2gene pipeline. Subsequently, as the variants are linked to genes through these analyses, the functions enriched within the gene set can be explored to identify potential dysregulated pathways relevant to the disease.

In this context we present Genopyc, a Python library for investigating the functional effects of variants associated with complex diseases. Genopyc allows users to programmatically access multiple sources with the aim of understanding how noncoding variants could impact the biological pathways and thus infer the mechanisms underlying the development of complex diseases (Fig. 1). Moreover, being fully integrated in Python allows to perform further analysis in the same environment using the most common packages.

## 2 Implementation and features

Genopyc is a Python package integrating information from several knowledge bases. The tool can receive as an input a trait, coded with Experimental Factor Ontology (EFO) identifiers (Malone *et al.* 2010), or the results of a GWA study. If an EFO code is used as an input, the variants associated to the trait are retrieved from the GWAS Catalog. Information such as the _**β**_ coefficient, standard error, risk allele frequency and the mapped genes related to the SNPs are also retrieved. Additionally, other features such as genomic coordinates, linkage disequilibrium (LD) correlated SNPs and neighboring functional elements can be obtained from Ensembl Genome Browser (Martin *et al.* 2023). Genopyc also queries the variant effect predictor (VEP) to obtain the consequences of the SNPs on the transcript and its effect on neighboring genes and functional elements (McLaren *et al.* 2016). Often SNPs associated to complex phenotypes fall in noncoding regions of the genome and are more likely to have regulatory effects (Prokunina and Alarcón-Riquelme 2004). Therefore, it is possible to retrieve the expression quantitative trait loci (eQTL) related to variants through the eQTL Catalogue (Kerimov *et al.* 2021). Finally, Genopyc integrates the locus to gene (L2G) pipeline from Open Target Genetics to uncover the target gene or genes of variants located in noncoding regions. Once a variant is associated to a gene or genes, the significantly enriched pathways are retrieved through G: Profiler (Raudvere *et al.* 2019). In this way the user can elucidate the functions whose perturbation could ultimately lead to the disease. Genopyc package also offers functionality to visualize the results of the functional enrichment as an interactive network (see Supplementary material). In this network, genes of interest are mapped to a protein-protein interaction network derived from the HIPPIE database (Alanis-Lobato *et al.* 2017) in which nodes represent the gene products and edges correspond to the physical interactions between proteins. A dropdown menu allows the user to select the function enriched in the gene set and, when a function is selected, the gene-products belonging to that function are highlighted.

Genopyc can also retrieve a linkage-disequilibrium (LD) matrix for a set of SNPs by using LDlink (Machiela and Chanock 2015), convert genome coordinates between genome versions and retrieve genes coordinates in the genome. LDlink calculates the LD matrix through the population-specific 1000 genomes haplotype panels (Auton *et al.* 2015). Retrieving genome coordinates and mapping between genome builds are made possible by accessing Ensembl genome browser. A comparison between the main functionalities of Genopyc and other tools for post-GWAS analysis is shown in Table 1. Genopyc is the only library that integrates multiple analysis to connect variants to genes (conditional, colocalization, fine mapping) through L2G pipeline, gather functional information to annotate variants (eQTLs, HI-C, linkage disequilibrium, VEP, functional genomic elements), maps between different vocabularies of gene and variant identifiers and perform functional enrichment to detect possible pathways perturbed by genetic variations. The visualization capabilities of the library help the user to directly unveil biological associations and can be fully exploited in an interactive computational environment such as Jupyter Notebook. In summary, we provide an all-in-one tool to retrieve and interpret the effect of genomic variants on the development of complex disease. Genopyc is easily installable via pip and can be integrated into Python environments being built upon main Python libraries.

**Table 1.** Comparison between Genopyc and the main tools for post GWAS analysis.^a^

Tool	Mapping Ids	Retrieve trait associated variants	Conditional analysis	Fine mapping	eQTL Co-localization	HI-C	Functional enrichment	Linkage disequilibrium	Genomic features	Variant annotation
Genopyc	_**✓**_	_**✓**_	_**✓**_	_**✓**_	_**✓**_	_**✓**_	_**✓**_	_**✓**_	_**✓**_	_**✓**_
Coloc	_**×**_	_**×**_	_**×**_	_**×**_	_**✓**_	_**×**_	_**×**_	_**×**_	_**×**_	_**✓**_
FUMA	_**×**_	_**×**_	_**✓**_	_**×**_	_**✓**_	_**✓**_	_**×**_	_**✓**_	_**×**_	_**×**_
Finemap	_**×**_	_**×**_	_**×**_	_**✓**_	_**×**_	_**×**_	_**×**_	_**✓**_	_**×**_	_**×**_
Ensemble API	_**✓**_	_**×**_	_**×**_	_**×**_	_**×**_	_**×**_	_**×**_	_**✓**_	_**✓**_	_**✓**_
Open targets: genetics	_**✓**_	_**✓**_	_**✓**_	_**✓**_	_**✓**_	_**✓**_	_**×**_	_**✓**_	_**×**_	_**✓**_

a Genopyc integrates diverse functionalities allowing a more flexible investigation of variants related to diseases.

## 3 Use case

To illustrate the utility of Genopyc, we applied it to the variants associated to lumbar disc degeneration that are available in the GWAS catalog [intervertebral disc degeneration (IDD), EFO:0004994]. IDD is a complex multifactorial condition for which the molecular mechanisms are poorly understood. Thanks to multiple data integration via Genopyc we highlight the involvement of variants associated downstream to pathways that may be relevant to the IDD, such as SP1 (Xu *et al.* 2016), HIF1-_**α**_ (Meng *et al.* 2018) and AP-2_**α**_ (Li *et al.* 2020), that according to the literature are tightly associated with IDD. Conversely, the functional enrichment did not bring any result or valuable information on the pathways that could be dysregulated in the disease. This example highlights that thanks to Genopyc a user can unveil a greater understanding of human complex traits.

## Supplementary Material

btae379_Supplementary_Data

## Data Availability

The library can be installed via pip: https://pypi.org/project/genopyc/. The source code is available at: https://github.com/freh-g/genopyc. The notebook with the use case is available at: https://github.com/freh-g/genopyc/blob/main/tutorials/Genopyc_tutorial_notebook.ipynb. The documentation of the package is available at: https://genopyc.readthedocs.io/en/latest/.

## References

[btae379-B1] Alanis-Lobato G , Andrade-Navarro MA, Schaefer MH. HIPPIE v2.0: enhancing meaningfulness and reliability of protein-protein interaction networks. Nucleic Acids Res 2017;45:D408–14. 27794551 10.1093/nar/gkw985 PMC5210659

[btae379-B2] Auton A , Brooks LD, Durbin RM et al; 1000 Genomes Project Consortium. A global reference for human genetic variation. Nature 2015;526:68–74. 26432245 10.1038/nature15393 PMC4750478

[btae379-B3] Bovonratwet P , Kulm S, Kolin DA et al Identification of novel genetic markers for the risk of spinal pathologies: a genome-wide association study of 2 biobanks. JBJS 2023;105. 10.2106/JBJS.22.00872 36927824

[btae379-B4] Bush WS , Moore JH. Chapter 11: genome-wide association studies. PLoS Comput Biol 2012;8:e1002822. 23300413 10.1371/journal.pcbi.1002822 PMC3531285

[btae379-B5] de Souza N. Genomics: the ENCODE project. Nat Methods 2012;9:1046. 23281567 10.1038/nmeth.2238

[btae379-B6] Edgar R , Domrachev M, Lash AE. Gene expression omnibus: NCBI gene expression and hybridization array data repository. Nucleic Acids Res 2002;30(1):207–10. 10.1093/nar/30.1.207 PMC99122 11752295

[btae379-B7] Edwards SL , Beesley J, French JD et al Beyond GWASs: illuminating the dark road from association to function. Am J Hum Genet 2013;93:779–97. 24210251 10.1016/j.ajhg.2013.10.012 PMC3824120

[btae379-B8] Gallagher MD , Chen-Plotkin AS. The Post-GWAS era: from association to function. Am J Hum Genet 2018;102:717–30. 29727686 10.1016/j.ajhg.2018.04.002 PMC5986732

[btae379-B9] Kerimov N , Hayhurst JD, Peikova K et al A compendium of uniformly processed human gene expression and splicing quantitative trait loci. Nat Genet 2021;53:1290–9. 34493866 10.1038/s41588-021-00924-w PMC8423625

[btae379-B10] Li B , Ritchie MD. From GWAS to gene: transcriptome-wide association studies and other methods to functionally understand GWAS discoveries. Front Genet 2021;12:713230. 34659337 10.3389/fgene.2021.713230 PMC8515949

[btae379-B11] Li H , Li W, Liang B et al Role of AP-2α/TGF-β1/Smad3 axis in rats with intervertebral disc degeneration. Life Sci 2020;263:118567. 33038379 10.1016/j.lfs.2020.118567

[btae379-B12] Lonsdale J , Thomas J, Salvatore M et al; GTEx Consortium. The genotype-tissue expression (GTEx) project. Nat Genet 2013;45:580–5. 23715323 10.1038/ng.2653 PMC4010069

[btae379-B13] Machiela MJ , Chanock SJ. LDlink: a web-based application for exploring population-specific haplotype structure and linking correlated alleles of possible functional variants. Bioinformatics 2015;31:3555–7. 26139635 10.1093/bioinformatics/btv402 PMC4626747

[btae379-B14] Malone J , Holloway E, Adamusiak T et al Modeling sample variables with an experimental factor ontology. Bioinformatics 2010;26:1112–8. 20200009 10.1093/bioinformatics/btq099 PMC2853691

[btae379-B15] Martin FJ , Amode MR, Aneja A et al Ensembl 2023. Nucleic Acids Res 2023;51:D933–41. 36318249 10.1093/nar/gkac958 PMC9825606

[btae379-B16] McLaren W , Gil L, Hunt SE et al The Ensembl variant effect predictor. Genome Biol 2016;17:122. 27268795 10.1186/s13059-016-0974-4 PMC4893825

[btae379-B17] Meng X , Zhuang L, Wang J et al Hypoxia-inducible factor (HIF)-1alpha knockout accelerates intervertebral disc degeneration in mice. Int J Clin Exp Pathol 2018;11:548–57. 31938140 PMC6957989

[btae379-B18] Mountjoy E , Schmidt EM, Carmona M et al An open approach to systematically prioritize causal variants and genes at all published human GWAS trait-associated loci. Nat Genet 2021;53:1527–33. 34711957 10.1038/s41588-021-00945-5 PMC7611956

[btae379-B19] Mulder N , Opap K. Recent advances in predicting gene-disease associations. F1000Res 2017;6:578. 28529714 10.12688/f1000research.10788.1 PMC5414807

[btae379-B20] Pérez-Granado J , Piñero J, Furlong LI. Benchmarking post-GWAS analysis tools in major depression: challenges and implications. Front Genet 2022;13:1006903. 36276939 10.3389/fgene.2022.1006903 PMC9579284

[btae379-B21] Piñero J , Ramírez-Anguita JM, Saüch-Pitarch J et al The DisGeNET knowledge platform for disease genomics: 2019 update. Nucleic Acids Res 2019;48:D845–55. 10.1093/nar/gkz1021 PMC7145631 31680165

[btae379-B22] Prokunina L , Alarcón-Riquelme ME. Regulatory SNPs in complex diseases: their identification and functional validation. Expert Rev Mol Med 2004;6:1. 10.1017/S1462399404007690 15122975

[btae379-B23] Raudvere U , Kolberg L, Kuzmin I et al g: profiler: a web server for functional enrichment analysis and conversions of gene lists (2019 update). Nucleic Acids Res 2019;47:W191–8. 31066453 10.1093/nar/gkz369 PMC6602461

[btae379-B24] Sollis E , Mosaku A, Abid A et al The NHGRI-EBI GWAS catalog: knowledgebase and deposition resource. Nucleic Acids Res 2023;51:D977–85. 36350656 10.1093/nar/gkac1010 PMC9825413

[btae379-B26] Uffelmann E , Huang QQ, Munung NS et al Genome-wide association studies. Nat Rev Methods Primers 2021;1:1063–77.

[btae379-B28] Xu K , Wang X, Zhang Q et al Sp1 downregulates proinflammatory cytokine-induced catabolic gene expression in nucleus pulposus cells. Mol Med Rep 2016;14:3961–8. 27600876 10.3892/mmr.2016.5730

